# TRPV4 Complexes With the Na^+^/Ca^2+^ Exchanger and IP_3_ Receptor 1 to Regulate Local Intracellular Calcium and Tracheal Tension in Mice

**DOI:** 10.3389/fphys.2019.01471

**Published:** 2019-12-06

**Authors:** Jie Zhang, Yuan Wei, Suwen Bai, Shenggang Ding, Huiwen Gao, Sheng Yin, Shuo Chen, Jinsen Lu, Haoran Wang, Yonggang Shen, Bing Shen, Juan Du

**Affiliations:** ^1^School of Basic Medical Sciences, Anhui Medical University, Hefei, China; ^2^Department of Physiology, College of Basic Medical Sciences, Army Medical University, Chongqing, China; ^3^Department of Paediatrics, The First Affiliated Hospital of Anhui Medical University, Hefei, China; ^4^Department of Neurosurgery, Anhui Provincial Hospital, Anhui Medical University, Hefei, China; ^5^Nursing Faculty, Anhui Health College, Chizhou, China

**Keywords:** transient receptor potential vanilloid 4, Na^+^/Ca^2+^ exchanger, IP_3_ receptor, airway smooth muscle, carbachol

## Abstract

Intracellular Ca^2+^ is critical for regulating airway smooth muscle (ASM) tension. A rapid rise in the intracellular Ca^2+^ concentration ([Ca^2+^]_i_) of ASM cells is crucial for modulating the intensity and length of the ASM contraction. Because this rapid increase in [Ca^2+^]_i_ largely depends on the balance between Ca^2+^ released from intracellular Ca^2+^ stores and extracellular Ca^2+^ entry, exploring the mechanisms mediating Ca^2+^ transport is critical for understanding ASM contractility and the pathogenesis of bronchial contraction disorders. Transient receptor potential vanilloid 4 (TRPV4) is a highly Ca^2+^-permeable non-selective cation channel that mediates Ca^2+^ influx to increase [Ca^2+^]_i_, which then directly or indirectly regulates the contraction and relaxation of ASM. The [Ca^2+^]_i_ returns to basal levels through several uptake and extrusion pumps, such as the sarco(endo)plasmic reticulum Ca^2+^ ATPase and inositol 1,4,5-trisphosphate receptors (IP_3_Rs), the plasmalemmal Ca^2+^ ATPase, and the plasma membrane Na^+^/Ca^2+^ exchanger (NCX). Thus, to further understand ASM tension regulation in normal and diseased tissue, the present study examined whether an interaction exists among TRPV4, IP_3_Rs, and NCX. The TRPV4-specific and potent agonist GSK1016790A increased [Ca^2+^]_i_ in mouse ASM cells, an effect that was completely blocked by the TRPV4-specific antagonist HC067047. However, GSK1016790A induced relaxation in mouse tracheal rings precontracted with carbachol *in vitro*. To determine the mechanism underlying this TRPV4-induced relaxation of ASM, we blocked specific downstream molecules. We found that the GSK1016790A-induced relaxation was abolished by the NCX inhibitors KB-R7943 and LiCl but not by specific inhibitors of the Ca^2+^-activated large-, intermediate-, or small-conductance K^+^ channels (BK_Ca_, IK, and SK_3_, respectively). The results of co-immunoprecipitation (co-IP) assays showed an interaction of TRPV4 and IP_3_R_1_ with NCX_s_. Taken together, these findings support a physical and functional interaction of TRPV4 and IP_3_R_1_ with NCXs as a novel TRPV4-mediated Ca^2+^ signaling mechanism and suggest a potential target for regulation of ASM tension and treatment of respiratory diseases, especially tracheal spasm.

## Introduction

Control of the intracellular calcium concentration ([Ca^2+^]_i_) is critical to the regulation of airway smooth muscle (ASM) function and mediates many processes, including contraction, proliferation, and gene expression (Hirota and Janssen, 2007). Contractility in ASM is largely mediated by the interaction of ligands with specific G protein-coupled receptors (GPCRs), leading to Ca^2+^ release from intracellular stores as well as Ca^2+^ influx from the extracellular medium. The transient receptor potential vanilloid 4 (TRPV4) channel is a subclass of the TRPV ion channel family (Ferreira and Faria, 2016). TRPV4 is a highly Ca^2+^-permeable, non-selective cation channel (Grace et al., 2017). The TRPV4 channel can be activated by various stimulating factors, including moderate heat (>24°C), osmotic pressure changes, shear stress, cell swelling, and chemical stimulation (Watanabe et al., 2003; Grace et al., 2017). TRPV4 channels are widely expressed in various tissues, including ASM (Jia et al., 2004). In the ASM of humans and guinea pigs, activation of TRPV4 by relevant stimulators mediates Ca^2+^ influx and increases [Ca^2+^]_i_ (Jia et al., 2004; Allen et al., 2014; Buday et al., 2017). The Ca^2+^ rise in the ASM comes from two primary sources: channels in the plasma membrane that mediate Ca^2+^ entry into the cell, and internal stores sequestered in the endoplasmic reticulum (ER) or sarcoplasmic reticulum (SR) that mediate intracellular Ca^2+^ release. Junctional membrane complexes between the plasma membrane and the ER/SR are needed to provide effective mechanisms for cross talk between Ca^2+^ channels/transporters in the plasma membrane and Ca^2+^-release channels in intracellular membranes (Ma and Pan, 2003; Maack and O’Rourke, 2007; Yamazaki et al., 2009). In addition, TRPV4 and the inositol 1,4,5-trisphosphate (IP_3_) receptors have a direct association, and IP_3_ via its receptors potentiates TRPV4 sensitivity to the mechano- and osmo-transducing messenger 5′-6′-epoxyeicosatrienoic acid (Jacqueline et al., 2008).

After the effects of an agonist, intracellular Ca^2+^ returns to basal levels through several uptake and extrusion pumps, such as the ER/SR Ca^2+^ ATPase, plasmalemmal Ca^2+^ ATPase, and plasma membrane Na^+^/Ca^2+^ exchanger (NCX) (Janssen et al., 1997; Blaustein and Lederer, 1999). The plasma membrane NCX is a bidirectional transporter that catalyzes electrogenic exchange of three Na^+^ ions for one Ca^2+^ ion depending on the electrical and chemical gradients of the subregion (Matsuda et al., 1997; Blaustein and Lederer, 1999). This exchanger plays a critical role in regulating [Ca^2+^]_i_ by primarily exporting Ca^2+^ to the outside of the cell. Although in the forward mode of operation, NCX mediates Ca^2+^ efflux and Na^+^ influx, in the reverse mode of operation, NCX mediates Ca^2+^ influx and Na^+^ efflux. In the forward mode, NCX operates in parallel with the plasma membrane Ca^2+^-ATPase pump to maintain Ca^2+^ homeostasis. The mammalian NCX comprises three isoforms: NCX1, NCX2, and NCX3 (Giladi et al., 2016; Grover, 2017). The expression, function, and regulation of NCX differ across tissues and species (Blaustein and Lederer, 1999). The results from diverse types of studies indicate that NCX plays an important role in Ca^2+^ influx and efflux in smooth muscle (Blaustein and Lederer, 1999). For example, NCX-mediated Ca^2+^ flux contributes to myogenic vasoconstriction in rat cremaster muscle arterioles (Sakai et al., 2005), and NCX-mediated Ca^2+^ efflux appears to be important for [Ca^2+^]_i_ homeostasis in guinea pig stomach (Raina et al., 2008). The physical proximity of NCX to the perimembranous SR and a relationship between NCX and the canonical transient receptor potential channel proteins (Christian et al., 2004; Zhang et al., 2005) provide further supporting evidence for a role of NCX in [Ca^2+^]_i_ homeostasis.

To rapidly and efficiently activate downstream signaling molecules, several molecules involved in the same signal transduction form signal complexes. In the present study, we evaluated whether an interaction occurred for TRPV4, the IP_3_ receptor (IP_3_R), and NCX and the potential functions of such an interaction in ASM contraction. The discovery of such an interaction would further understand ASM tension regulation and may contribute to the development of compounds effective in the treatment of respiratory diseases, especially tracheal spasm.

## Materials and Methods

### Chemicals

Carbachol, GSK1016970A, KB-R7943, HC067047, apamin, and lithium chloride (LiCl) were purchased from Sigma (United States). Iberiotoxin (IbTX), and TRAM34 were purchased from MedChemExpress. The anti-TRPV4 antibody, anti-IP_3_R_1_ antibody, and KB-R7943 were purchased from Santa Cruz Biotechnology. The anti-NCX_1_ antibody, anti-NCX_2_ antibody, and anti-NCX_3_ antibody were purchased from the Beijing Bioss company.

### Animals and Ethical Standards

Male Kunming mice (18–20 g) were obtained from the Experimental Animal Center of Anhui Medical University and used in accordance with guidelines of the U.S. National Institutes of Health (NIH publication No. 8523). This study was approved by the Animal Experimentation Ethics Committee of Anhui Medical University. Mice were housed in a facility with a 12-h light-dark cycle, a temperature of 24°C, and humidity of 60% and were allowed food and water *ad libitum*. The equations should be inserted in editable format from the equation editor.

### Primary Culture of Mouse ASM Cells

Mouse ASM cells were primary cultured through enzyme digestion as previously reported (Ding et al., 2019). Briefly, tracheas were isolated and placed in ice-cold Krebs–Henseleit solution (composition in mM: NaCl 118, NaHCO_3_ 25.2, KCl 4.7, CaCl_2_ 2.5, glucose 11.1, KH_2_PO_4_ 1.2, and MgSO_4_.7H_2_O 1.2). After the epithelial layer was rubbed off, the trachea was digested with 0.2% collagenase type IA and 0.9% papain for 60 h. The dispersed tracheal smooth muscle cells were cultured in Dulbecco’s modified Eagle’s medium containing 10% fetal bovine serum, 100 μg/mL streptomycin and 100 U/mL penicillin at 37°C. In order to remove non-adherent cells, the medium was replaced after a 60 h incubation. The remaining adherent ASM cells were cultured at 37°C with 5% CO_2_ for 5–7 days before experimental use. The ASM marker α-SMA and fibroblasts marker FSP are used to characterize the specificity of ASM cells (Supplementary Figures 1A–C).

### Hematoxylin and Eosin Staining

Airway of mice were immersed in 4% neutral formaldehyde for 48 h. The airway tissues were processed routinely for paraffin embedding, and 4-μm-thick sections were cut and placed on glass slides. Tissue samples were blocked with 5% bovine serum albumin (BSA), and then incubated with primary antibodies against TRPV4 (Affinity, 1:250) followed by incubation with a goat anti-rabbit secondary antibody. Images were acquired by microscopy (Olympus, Tokyo, Japan).

### Measurement of Intracellular Ca^2+^ Concentration

The [Ca^2+^]_i_ was measured as previously described (Du et al., 2012). Briefly, ASM cells were incubated with 6 μmol/L Fluo-8 AM (a medium affinity green fluorescent Ca^2+^ binding dye) and 0.02% pluronic F-127 (Invitrogen) for 30 min. The cells were treated with 1 nM GSK1016790A with or without 10 μM HC067047. Fluorescence was recorded using a fluorescence microscope (Nikon, Tokyo, Japan) having a xenon lamp with excitation and emission wavelengths of 488 nm. The [Ca^2+^]_i_ changes are presented as the ratio of the experimental fluorescence relative to the initial fluorescence intensity (*F*_1_/*F*_0_).

### Measurement of Contractile Tension

Mice were humanely killed with CO_2_, and the trachea was isolated and placed in a container filled with ice-cold Krebs–Henseleit solution oxygenated with a mixture of 95% O_2_ and 5% CO_2_. After rubbing off the epithelial layer by gentle friction with minimal damage to the smooth muscle, the trachea was cut into 2–3 mm segments. The tracheal rings were transferred to an organ bath containing Krebs–Henseleit solution (pH 7.4) saturated with 95% O_2_ plus 5% CO_2_ at 37°C. One end of the ring was fastened to a hook on the bottom of the organ bath using surgical silk suture. The other end was connected to an isometric force transducer that was connected to an integrated amplifier and recorder. The isometric tension was recorded and analyzed by a data acquisition and analysis system (BL-420S, Chengdu Taimeng Technology). In each experiment, the tracheal rings were stretched to a preload tension of 5 mN and equilibrated for 1 h, with a change of physiological buffer every 15 min and appropriate adjustment of tension. After achieving constant tracheal ring tension for at least 30 min, agonists with or without inhibitors were added. At the end of the experiment, the tracheal rings were dried and weighed. Responses were calculated as mg/mg tissue weight and in some cases are expressed as a percentage of the maximum response.

### Co-immunoprecipitation and Immunoblots

Co-immunoprecipitation (co-IP) assays were performed using previously described procedures (Du et al., 2012). Briefly, the smooth muscle layer was peeled from the adventitial layer of the trachea with forceps and homogenized. The smooth muscle cells were lysed with protein lysis buffer (1% Non-idet P-40, 150 mM NaCl, 20 mM Tris–HCl at pH 8.0, with the addition of a protease inhibitor cocktail), sonicated, and centrifuged at 10,000 × *g* for 15 min at 4°C. TRPV4, NCX1, NCX2, NCX3, or IP_3_R1 proteins were immunoprecipitated by incubating 800 μg extracted proteins with 5 μg anti-TRPV4, anti-NCX1, anti-NCX2, anti-NCX3, or anti-IP_3_R1 antibody or preimmune IgG on a rocking platform overnight at 4°C. Protein A agarose was then added and incubated for an additional 3 h at 4°C. The immunoprecipitates were washed three times with phosphate-buffered saline. For the immunoblots, all of the samples were fractionated separated using 7.5% sodium dodecyl sulfate polyacrylamide gel electrophoresis, transferred to poly (vinylidene difluoride) membranes, and probed with the indicated primary antibodies at 1:200 dilution in phosphate-buffered saline that contained 0.01% Tween 20 and 5% non-fat dry milk. Immunodetection was accomplished using a horseradish peroxidase–conjugated secondary antibody and an enhanced chemiluminescence detection system.

### Respiration Measurement

The mice were randomly divided into three groups: control group, TRPV4 activation group and TRPV4 inhibition group. The control group was aerosol inhalation with PBS + DMSO, the activation group was aerosol inhalation with GSK1016790A (10 nM), and the inhibition group was aerosol inhalation with HC067047 (10 μM). The respiratory measurement was done at 0, 30, 60, and 90 min after atomization. When measuring the breath of mice, the mice were individually placed into a typical breathing box with a respiration transducer connected to a biological data acquisition and analysis system (BL-420S, Chengdu Taimeng Technology) to collect and analyze the respiratory parameters of breathing rate. The screws of box were tighten to avoid air leaks, and a towel was placed around the box to keep the environment dark. The respiratory rate and amplitude were recorded for 10 min. Care was taken during the experiment to avoid generating noise and to avoid touching the box.

### Statistical Analysis

The experimental data are presented as the means ± SEM. The Mann–Whitney test (two-tailed) was conducted using GraphPad Prism 5 software (GraphPad Software, San Diego, CA, United States) to compare the results between groups. *P*-values < 0.05 were considered statistically significant.

## Results

### TRPV4 Effect on Respiration

**FIGURE 1:**
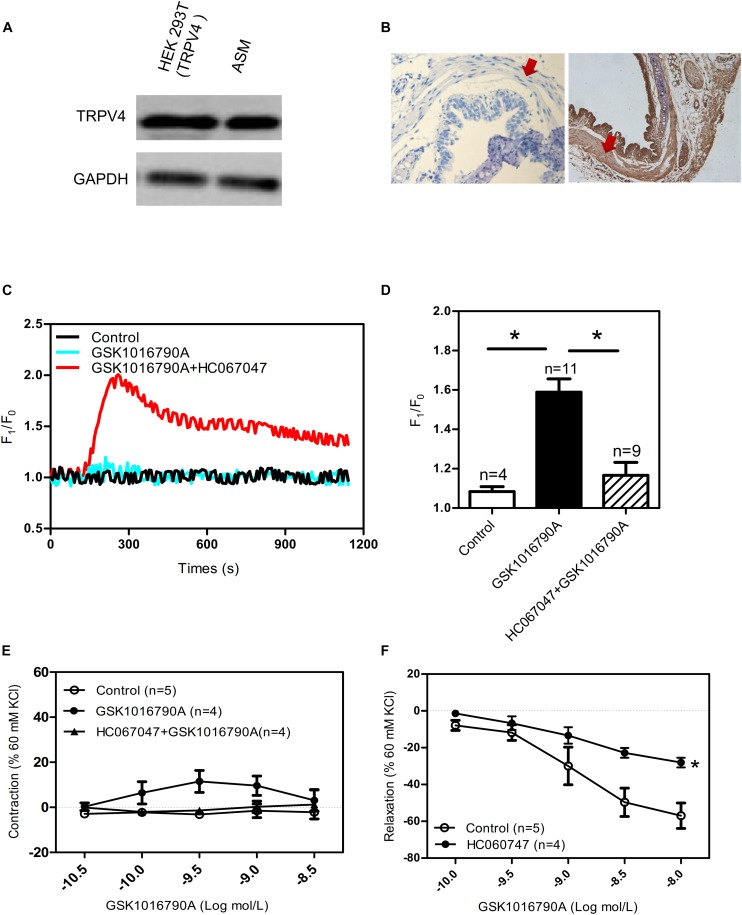
Functional expression of TRPV4 in mouse airway smooth muscle. **(A)** Western blot analysis shows TRPV4 expression in HEK293T cells with transiently expressed TRPV4 (positive control) and in mouse trachea smooth muscle cells. **(B)** Immunohistochemical results for TRPV4 expression in mouse. Negative control is incubated by preimmune IgG. The arrows showed smooth muscle. **(C,D)** Representative traces and summary data showing the TRPV4 activator 10 nM GSK1016790A-induced alterations in the [Ca^2+^]_i_ concentration in the presence or absence of 10 μM HC067047. Data are shown as means ± SE; *n* = 4–11. ^∗^*P* < 0.05, compared with GSK1016790A group. **(E)** Summary data showing GSK1016790A-induced tracheal contractions with or without HC067047. **(F)** Summary data showing carbachol precontracted-tracheal relaxation induced by the TRPV4 agonist GSK1016790A was significantly reduced by pretreatment with the TRPV4 inhibitor HC067047. Data are shown as means ± SE; *n* = 4–5. ^∗^*P* < 0.05, compared with control group.

As previously mentioned, the expression and function of TRPV4 in ASM differs across species. Consistent with studies that have reported functional expression of TRPV4 in human and guinea pig ASM (Jia et al., 2004; Allen et al., 2014; Buday et al., 2017), we found that TRPV4 was expressed by the experiments of western blotting and Hematoxylin and Eosin Staining in mouse ASM (Figures 1A,B). In addition, the specific TRPV4 agonist GSK1016790A (10 nM) induced a transient increase in [Ca^2+^]_i_ in primary cultured mouse ASM cells, and this response was completely abolished by HC067047 (10 μM), a TRPV4 blocker (Figures 1C,D). In experiments using tonically contracted tracheal rings, GSK1016790A barely induced further tracheal contraction (Figure 1E). However, activation of TRPV4 resulted in concentration-dependent relaxation in mouse tracheal rings precontracted by 1 mM carbachol, a muscarinic receptor agonist; the relaxation induced by a final cumulative concentration of 10 nM GSK1016790A was about 40%, and which was significantly weakened by pretreatment of 10 μM HC067047 (Figure 1F). The tension of tracheal rings are expressed as force normalized to high potassium contraction).

### Physical and Functional Association of TRPV4 and IP_3_R Regulates Tracheal Tension

**FIGURE 2:**
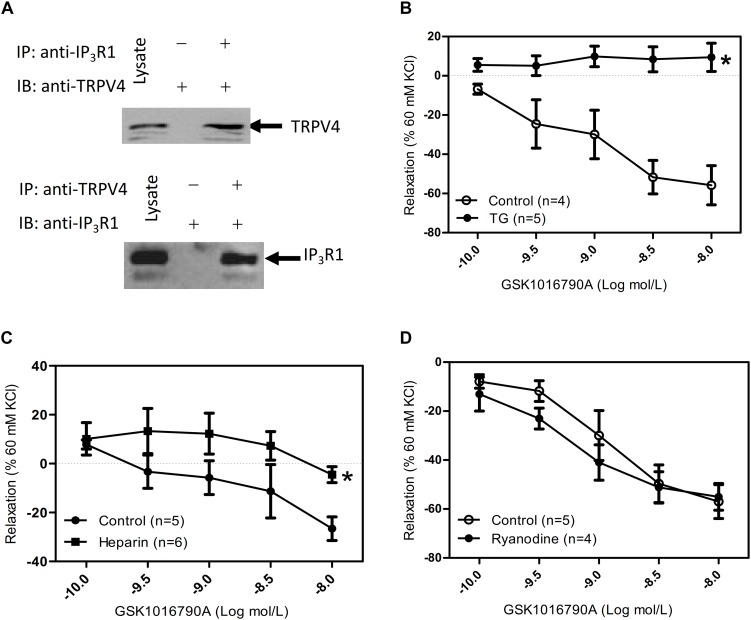
Physical and functional association of TRPV4 and Ca^2+^ channel in ER/SR. **(A)** TRPV4 and IP_3_R1 co-immunoprecipitate. The pulling antibody and the blotting antibody are indicated. Control immunoprecipitation was performed using preimmune IgG (labeled as – and +). IP represents immunoprecipitate and IB indicates immunoblot. **(B–D)** Summary data showed that 1 mM carbachol precontracted-tracheal relaxation induced by the TRPV4 agonist 10 nM GSK1016790A was significantly reduced by pretreatment with the TG (4 μM) or with heparin (150 mg μg/mL) **(B,C)**, but not by ryanodine (10 μM) **(D)**. Tracheal ring tension is expressed as force normalized to high-potassium (60 mM KCl) contractions. Data are shown as means ± SE; *n* = 4–6 mice. ^∗^*P* < 0.05, compared with control group.

In ASM cells, carbachol binds with GPCRs and mediates activation of phospholipase C to trigger IP_3_ production and activate the IP_3_ receptor to induce [Ca^2+^]_i_ rise (Ehlert, 2003). In addition, TRPV4 and the IP_3_ receptors have a direct association, and IP_3_ via its receptors potentiates TRPV4 sensitivity to the mechano- and osmo-transducing messenger 5′-6′-epoxyeicosatrienoic acid in endothelial cells (Jacqueline et al., 2008). Vertebrate genomes encode three IP_3_R subtypes (IP_3_R1-3). Each forms a Ca^2+^ channel that is co-regulated by IP_3_ and Ca^2+^, but the subtypes differ in their expression patterns (Taylor et al., 1999), affinities for IP_3_ (Miwako et al., 2007), and modulation by additional signals (Prole and Taylor, 2016). Relative to IP_3_R2 and IP_3_R3, IP_3_R1 is the predominant subtype expressed in ASM cells (Song et al., 2015). To reveal whether cross talk occurs between TRPV4 and IP_3_ receptor, we used co-IP assays. We found that TRPV4 could be pulled down with an anti-IP_3_R1 antibody, and conversely, that IP_3_R1 could be pulled down with anti-TRPV4 antibody (Figure 2A). In the results of tracheal tension experiment, GSK1016790A-induced tracheal ring relaxation in 1 mM carbachol-preconstricted treachea was blocked by the endoplasmic Ca^2+^-ATPase blocker thapsigargin (TG) (4 μM) or the IP_3_ receptor inhibitor heparin (150 μg/mL), but could not inhibit by raynodine receptor (RYR) inhibitor raynodine (10 μM) (Figures 2A–D). These results suggested that through the physical association of TRPV4 and Ca^2+^ rise mediated by IP_3_R1 to rapidly and accurately activate the TRPV4 and then induce activation of downstream signaling pathway to cause ASM relaxation.

### Functional Association of TRPV4 and NCX Regulates Tracheal Tension

**FIGURE 3:**
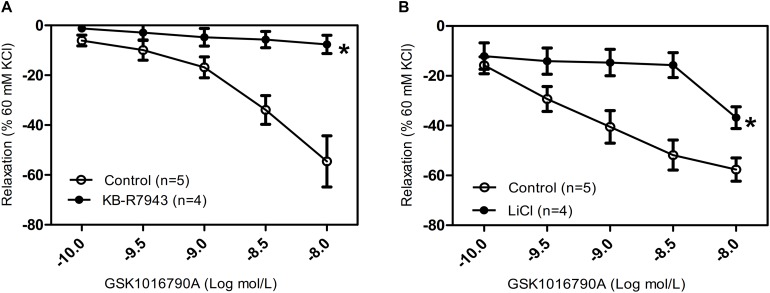
Effects of NCX inhibitors on tracheal rings precontracted with a TRPV4 agonist. **(A,B)** Compared with that in the control groups, carbachol precontracted-tracheal relaxation induced by the TRPV4 agonist GSK1016790A was significantly reduced by pretreatment with the NCX inhibitor KB-R7943 (5 μM) or with LiCl (118 mM). Tracheal ring tension is expressed as force normalized to high-potassium (60 mM KCl) contractions. Data are shown as means ± SE; *n* = 4–5 mice. ^∗^*P* < 0.05, compared with control group.

The TRPV4-induced intracellular Ca^2+^ increase resulted in relaxation of tracheal rings that were precontracted with 1 mM carbachol, suggesting that activation of TRPV4 may excite different downstream signaling pathways dependent on the local Ca^2+^ concentration. NCX has an important role in regulating intracellular Ca^2+^ homeostasis. In order to assess whether NCX is activated by the TRPV4-induced increase in local Ca^2+^ concentration, we used the specific NCX inhibitor KB-R7943 (5 μM) and Krebs–Henseleit solution that NaCl was substituted by LiCl (118 mM). We found that compared with the response in the control group, pretreatment with 5 μM KB-R7943 or with LiCl significantly inhibited the GSK1016790A-induced tracheal ring relaxation (Figures 3A,B). These results suggested that with an increasing carbachol-induced whole cell Ca^2+^ concentration, the TRPV4-induced local Ca^2+^ rise in turn activated the nearby NCX to export Ca^2+^ to the outside of the cell, thereby decreasing the free Ca^2+^ concentration and eliciting tracheal ring relaxation.

### No Functional Association of TRPV4 and Ca^2+^-Activated K^+^ Channels in the Regulation of Tracheal Tension

**FIGURE 4:**
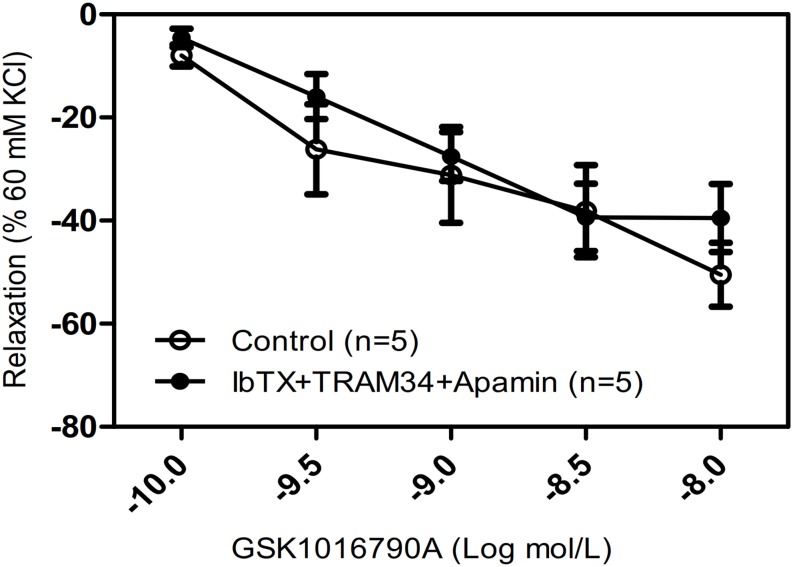
Effects of Ca^2+^-activated K^+^ channel inhibitors on tracheal rings precontracted with a TRPV4 activator. Compared with that in the control groups, the carbachol precontracted-tracheal ring relaxation-induced by GSK1016790A was not significantly reduced by pretreatment with the large-, intermediate-, and small-conductance Ca^2+^-activated K^+^ channel inhibitors IbTX (50 μM), TRAM34 (10 μM), and apamin (1 μM). Data are shown as means ± SE; *n* = 5 mice.

Hyperpolarization due to stimulation of large-conductance Ca^2+^-activated K^+^ channels (BKCa) is another potential mechanism for Ca^2+^-mediated relaxation in ASM cells. To determine whether the observed relaxation was due to TRPV4-mediated Ca^2+^ activation of BK_Ca_, we pretreated mouse tracheal rings with the specific BK_Ca_ blocker IbTX. Our results showed that the pretreatment of IbTX (50 μM), even in the presence of the intermediate-conductance Ca^2+^-activated K^+^ (IK) channel blocker TRAM34 (10 μM) and the small-conductance Ca^2+^-activated K^+^ (SK) channel blocker apamin (1 μM), had no effect on the TRPV4-induced relaxation of carbachol-induced precontracted tracheal rings (Figure 4).

### Physical Association of TRPV4, NCX, and IP_3_R1

**FIGURE 5:**
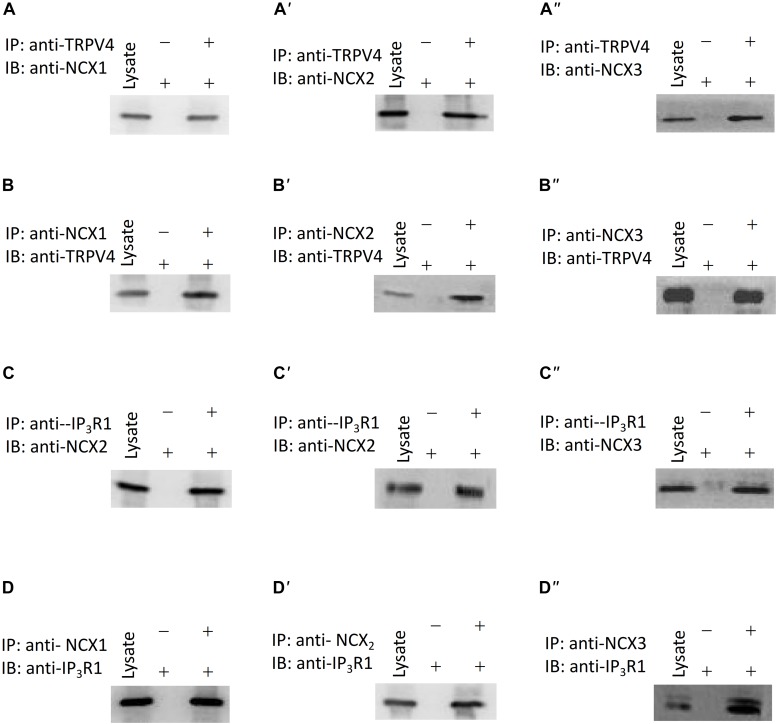
Interaction of TRPV4, NCXs, and IP_3_R1. **(A–A”,B–B”)** TRPV4 and NCX co-immunoprecipitate **(C–C”,D–D”)**. IP_3_R1 and NCX co-immunoprecipitate. The pulling antibody and the blotting antibody are indicated. Control immunoprecipitation was performed using preimmune IgG (labeled as – and +). IP represents immunoprecipitate and IB indicates immunoblot.

In order to rapidly and accurately regulate physiological processes, signaling components are located near one another and may interact to form complexes. There are three subtypes of NCX: NCX1, NCX2, and NCX3. The NCX is encoded by three gene isoforms, which generate NCX1, -2, and -3, NCX1 is a major isoform of NCXs in human ASM cells (Bo et al., 2010). Our western blotting results showed that NCX1, -2, and -3 expressed in murine ASM cells, and the expression level of NCX2 is higher than NCX1, 3 (Supplementary Figures 2A,B). To test for a potential physical association between TRPV4 and these NCX proteins, we performed co-IP studies using mouse ASM. We found that an anti-TRPV4 antibody could pull down NCX1,2,3, and conversely, the anti-NCX1,2,3 antibody could pull down TRPV4 (Figures 5A,B). These results provided clear evidence for a specific association of TRPV4 and NCX and suggested that they may form a functional complex in mouse ASM. To further reveal whether cross talk occurs between the TRPV4/NCX Ca^2+^ channel/transporter complex in the plasma membrane and the IP_3_ receptor, which is an IP_3_-sensitive Ca^2+^ channel on intracellular membranes that releases Ca^2+^ from intracellular stores, we again used co-IP assays. We found that TRPV4 and also NCX1,2,3 could be pulled down with an anti-IP_3_R1 antibody, and conversely, that IP_3_R1 could be pulled down with anti-TRPV4 and anti-NCX1,2,3 antibodies (Figures 5C,D). These results suggested that TRPV4, IP_3_R1, and NCX form a complex in ASM.

### Respiratory Rate in Mice

**FIGURE 6:**
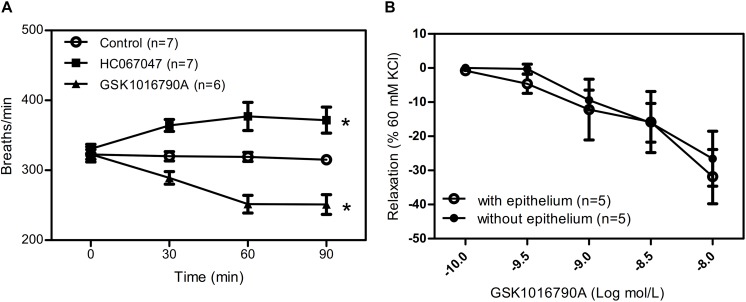
Role of TRPV4 agonist and inhibitor in mice respiratory rate. **(A)** Summary data showing respiratory rate in mice treated by aerosol inhalation of TRPV4 agonist GSK1016790A and TRPV4 inhibitor HC067047 and measure the breath rate of mice at 0, 30, 60, and 90 min of aerosol inhalation *in vivo*. **(B)** Summary data showing carbachol precontracted-tracheal relaxation induced by the TRPV4 agonist GSK1016790A with or without epithelium. Data are shown as means ± SE; *n* = 5–7 mice. ^∗^*P* < 0.05.

To further explore the role of TRPV4 *in vivo*, we used the aerosol inhalation of TRPV4 agonist GSK1016790A (10 nM) and TRPV4 inhibitor HC067047(10 μM) to mice and measure the breath rate of mice at 0, 30, 60, and 90 min of aerosol inhalation. The results showed that TRPV4-specific inhibitor HC067047 significantly increased the respiratory rate and the TRPV4 activator GSK1016790A significantly decreased the respiratory rate in time dependence (Figure 6A). In order to reveal the role of activation or inhibition of TRPV4 through smooth muscle directly or epithelial cells, we also measure the tension of trachea with or without epithelial cells induced by GSK1016790A (10 nM), and the results suggested that there are no difference between the two group (Figure 6B), which suggested that GSK1016790A or HC067047 regulate directly contraction of ASM cells in aerosol inhalation *in vivo*.

## Discussion

The major finding of this study was that TRPV4, IP_3_R1, and NCX2 interact to play important roles in the regulation of ASM tension and the respiratory rate in mice. The evidence supporting this statement follows. (1) Although the specific TRPV4 agonist GSK1016790A induced a transient [Ca^2+^]_i_ rise in primary cultured mouse ASM cells, this agonist also caused a concentration-dependent relaxation in mouse tracheal rings precontracted by carbachol. (2) Inhalation of TRPV4 blocker increased the respiratory rate of mice, whereas inhalation of the TRPV4 agonist decreased the rate. (3) The specific NCX inhibitor KB-R7943 and LiCl significantly inhibited the GSK1016790A-induced relaxation of tracheal rings precontracted with carbachol. (4) Pretreatment with the BK_Ca_ blocker IbTX, the IK blocker TRAM34, and the SK blocker apamin had no effect on the TRPV4-induced relaxation of tracheal rings precontracted with carbachol. (5) Co-IP assay analyses suggested that TRPV4, IP_3_R1, and NCX form a complex in ASM.

The contraction of ASM cells are modulated by the rapid [Ca^2+^]_i_ rise largely depends on the balance between Ca^2+^ releasing from intracellular Ca^2+^ stores and extracellular Ca^2+^ entry, exploring the mechanisms mediating Ca^2+^ transport is critical for understanding ASM contractility and the pathogenesis of bronchial contraction disorders. In ASM cells, carbachol binds with GPCRs and mediates activation of phospholipase C to trigger IP_3_ production and IP_3_-mediated Ca^2+^ release from the SR (Ehlert, 2003). Activation of the IP_3_R in the SR is critical for the Ca^2+^ rise induced by carbachol. The junctional membrane complexes between the plasma membrane and the ER/SR are needed to provide effective mechanisms for cross talk between Ca^2+^ channels/transporters in the plasma membrane and Ca^2+^-release channels in the intracellular membranes (Ma and Pan, 2003; Maack and O’Rourke, 2007; Yamazaki et al., 2009). TRPV4 and IP_3_ receptors have a direct association, and IP_3_ via its receptors potentiates TRPV4 sensitivity to the mechano- and osmo-transducing messenger 5′-6′-epoxyeicosatrienoic acid (Jacqueline et al., 2008). Our co-IP experiments showed that TRPV4 and IP_3_R1 physically interact in the mouse ASM cells. These results suggested that carbachol binds with GPCRs and induces IP_3_-mediated Ca^2+^ release from the SR, and TRPV4 in the plasma membrane is activated through the physical interaction with the IP_3_R in mice ASM cells.

Na^+^/Ca^2+^ exchanger is a membrane Ca^2+^-handling protein that introduces three Na^+^ ions to the cytoplasm, while extruding one Ca^2+^ when in its forward mode. By contrast, in its reverse mode, it introduces Ca^2+^ and extrudes Na^+^, both modes play a major role in intracellular Ca^2+^ homeostasis (Reyes-García et al., 2018). The plasma membrane NCXs are the important conponent for regulation of the cytosolic Ca^2+^ concentration through the extrusion of Ca^2+^ ions in exchange for three Na^+^ ions and vice versa depending on the stage of contraction (Verkhratsky et al., 2018). Our data showed that the TRPV4-mediated Ca^2+^ increase did not lead to contraction of tracheal rings. However, after carbachol induced an increase in [Ca^2+^]_i_, activation of TRPV4 caused an additional local Ca^2+^ rise, and this lead to the relaxation of the ASM which is blocked by the NCX inhibitor KB-R7943 or LiCl substituting NaCl in Krebs–Henseleit solution. The results of previous studies examining the functional roles of NCX in ASM appear conflicting, suggesting that the contribution of NCX in ASM may be species dependent. Among the three isoforms of NCX, NCX1 is expressed in human ASM and NCX1-mediated Ca^2+^ fluxes contribute to enhanced [Ca^2+^]_i_ regulation in airway inflammation (Venkatachalem et al., 2011). Our results suggested that NCX1,2,3 is expressed in the mouse airway.

In order to rapidly and efficiently activate downstream signaling molecules, several molecules involved in the same signal transduction may form signaling complexes. Regarding the subcellular distribution of NCX, the physical proximity of NCX to the perimembranous SR in vascular smooth muscle has been demonstrated, and other investigators have suggested that a similar situation occurs in ASM (Chalmers et al., 2007; Hirota and Janssen, 2007; Simon et al., 2007). A potential mechanism for protein interactions in the plasma membrane and SR is through caveolar expression, as has been suggested in studies examining porcine aortic endothelial cells. TRPV4 also interacts with caveolin-1 in endothelial cells (Rath et al., 2012). Our co-IP experiments showed that TRPV4 and IP_3_R1 physically interact with NCX1,2,3. These findings support the physical interaction of NCX, TRPV4, and IP_3_R1 between the plasma membrane and the SR membrane which induced that the local Ca^2+^ rise induced by TRPV4 actives rapidly the adjacent NCX and then lead to a Ca^2+^ efflux, and form a negative feedback to elicit trachea relaxation. We also tested whether Ca^2+^-activated K^+^ channels, including BK_Ca_, IK, and SK, played a role by using the specific blockers IbTX, TRAM34, and apamin, respectively. Our results showed that Ca^2+^-activated K^+^ channels do not participate in the TRPV4-mediated relaxation of tracheal rings precontracted by carbachol.

## Conclusion

**FIGURE 7:**
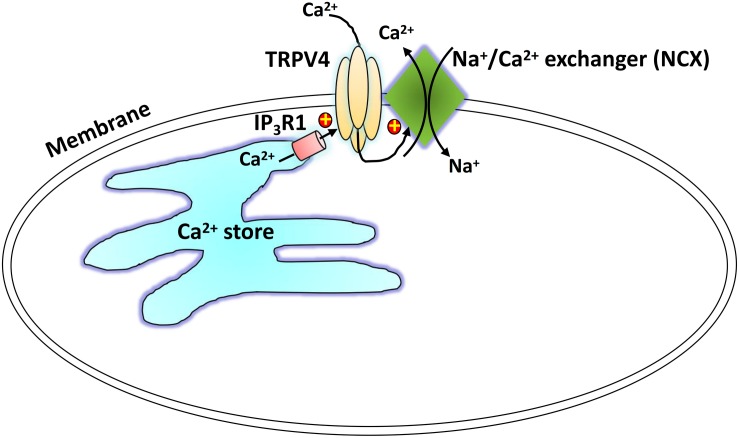
Schematic showing the proposed TRPV4-NCX-IP_3_R1 signaling complex transduction pathway. Carbachol binds with a G protein-coupled receptor to induce an IP_3_-mediated Ca^2+^ release from the sarcoplasmic reticulum. The physical interaction of the IP_3_ receptor activates a TRPV4-mediated increase in local Ca^2+^, which stimulates the adjacent NCX to induce Ca^2+^ efflux.

We suggest a physical and functional interaction of TRPV4 and IP_3_R1 with NCX as a novel regulatory mechanism underlying TRPV4-mediated Ca^2+^ signaling (Figure 7). Carbachol binds with GPCRs and induces IP_3_-mediated Ca^2+^ release from the SR, and TRPV4 in the plasma membrane is activated through the physical interaction with the IP_3_R. The local Ca^2+^ rise induced by TRPV4 actives the adjacent NCX to elicit to a Ca^2+^ efflux and relaxation of ASM cells. This TRPV4-IP_3_R1-NCX functional interaction achieves rapid signal transmission and regulation and contributes to ASM tension. This interaction suggests a potential target for regulation of ASM tension and treatment of certain respiratory diseases, especially tracheal spasm.

## Data Availability Statement

The raw data supporting the conclusion of this manuscript will be made available by the authors, without undue reservation, to any qualified researcher.

## Ethics Statement

The animal study was reviewed and approved by the Animal Experimentation Ethics Committee of Anhui Medical University.

## Author Contributions

JZ, YW, SB, SD, HG, SY, SC, JL, and HW developed the methodological aspects of analysis and data interpretation. JZ and SD analyzed the data, prepared the figures, and drafted the manuscript. JD drafted the manuscript. YW, SB, and HG contributed the extra experiments and followed reviewer’s suggestions. All authors revised the manuscript, approved the final version, and agreed to be accountable for all aspects of the work in ensuring that questions related to the accuracy or integrity of any part of the work are appropriately investigated and resolved.

## Conflict of Interest

The authors declare that the research was conducted in the absence of any commercial or financial relationships that could be construed as a potential conflict of interest.
