# Supplementary material for: TRPV4 Complexes With the Na+/Ca2+ Exchanger and IP3 Receptor 1 to Regulate Local Intracellular Calcium and Tracheal Tension in Mice
Source: Front Physiol. 2019 Dec 6;10:1471. doi: 10.3389/fphys.2019.01471 (PMC6910018; doi:10.3389/fphys.2019.01471)
Supplement: Supplementary file 1 [file Data_Sheet_1.PDF]

Supplemental Figure 1

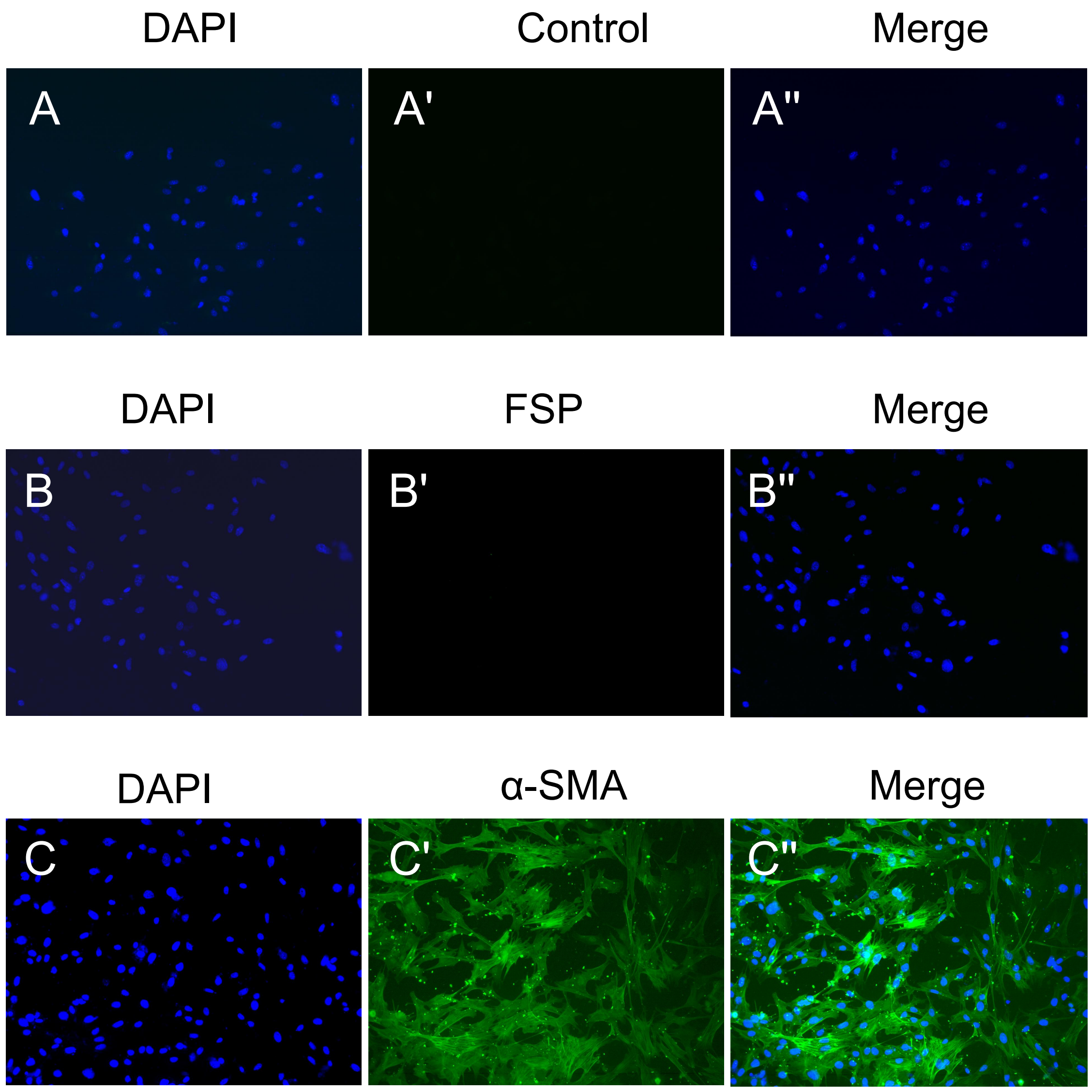

Supplemental Figure 1. Identify of primary cultured mouse ASM cells Primary ASM cells were stained with smooth muscle cell marker α-SMA or fibroblasts marker FSP antibody. In contrast to the control(omission of primary antibody)(A-A"), the staining for α-SMA was shown (B-B") and the staining for FSP was not shown in primary ASM cells (C-C"). Nuclei were stained by DAPI. Magnifcation is ×200.

Supplemental Figure 2

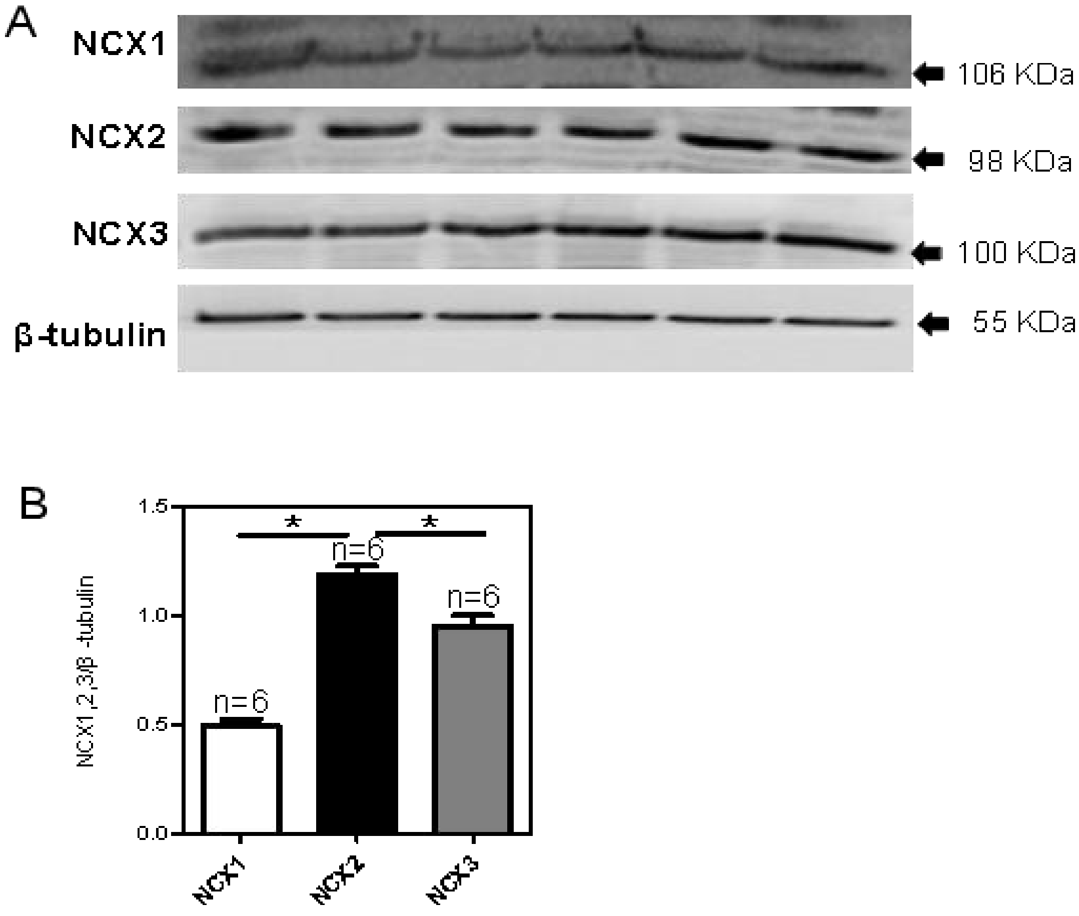

Supplemental Figure 2. Expression levels of NCXs in ASM cells from mice. Representative images (A) and summarized data (B) showing the expression levels of NCX1, NCX2, and NCX3 in primary cultured ASM cells from mice. β-tubulin was used as loading control. Protein levels were expressed as relative optical density. Data are shown as means ± S.E.; n =6. $*P < 0.05$, compared with NCX2.
